# A collaborative creation framework for experimental animation pedagogy integrating generative artificial intelligence and vibe coding

**DOI:** 10.3389/fpsyg.2026.1940505

**Published:** 2026-08-24

**Authors:** Yadan Mao

**Affiliations:** Department of Digital Media Art, School of Art, Soochow University, Suzhou, China

**Keywords:** animation pedagogy, cognitive load, experimental animation, generative artificial intelligence, human-AI collaboration, vibe coding

## Abstract

The growing use of generative artificial intelligence (GAI) in art and design has accelerated image and video automation. In this landscape, preserving media experimentation, authorial judgment, and generative unpredictability has emerged as a critical focus in experimental animation education. While existing studies mainly focus on production efficiency, limited attention has been paid to how GAI supports concept development, motion translation, media organization, and creative decision-making in classroom practice. Addressing this gap through design-based research (DBR), this study introduced GAI and Vibe Coding into a five-week undergraduate experimental animation course. It further proposed a human–AI co-creation framework based on “generation–feedback–evolution” and examined students’ creative processes and cognitive changes through process logs, creative portfolios, reflective texts, and three-stage questionnaires. The findings show that GAI extends far beyond mere production efficiency and actively intervenes in the decision-making process, prompting students to critically evaluate and revise their creative intentions, motion logic, and media expression. As human-AI interaction deepened, students moved from tool-based utility towards active selection, recombination, and transformation of AI-generated outcomes, reflecting a heightened awareness of their own creative agency. Questionnaire results indicate that, by the end of the course, students’ perception of AI as a collaborative partner and their germane load had both increased, extraneous load had decreased, and intrinsic load remained relatively stable. Qualitative analysis reveals that students began to engage in critical negotiations with AI regarding creative intentions, motion rules, media specificity, and authorial position. Preliminary findings suggest that the proposed framework can effectively support human-AI collaboration in experimental animation pedagogy, offering empirical, classroom-based evidence for understanding the shifting dynamics of authorship and media relations under AI intervention.

## 1 Introduction

Generative artificial intelligence is reshaping production mechanisms in the animation industry and challenging established models of animation education. Experimental animation has long served as a testing ground for emerging technologies. It engages with media convergence, the boundaries of artistic language, and new conceptual forms (Lu, 2021). Therefore, the central issue of introducing AI into experimental animation education is not merely tool training or efficiency. It is how creative intention, motion language, media reflection, and authorial judgment are reorganized under algorithmic generation.

In recent years, generative tools have been used in animation production and teaching for image style transfer, moving-image generation, and character design. However, most applications still treat AI as a tool for efficiency and material accumulation (Singh and Kaur, 2023), while paying less attention to its impact on conceptual development, creative judgment, and subject relations in experimental animation. For experimental animation, AI has a dual influence. Its dependence on existing data and algorithmic models threatens to produce stylistic homogenization and expressive mediocrity; however, its accidental, divergent, and unexpected outputs can also provide new clues for media experimentation. Given this dual effect, the focus of experimental pedagogy must shift toward guiding students to transform the generativity, variability, and iterability of AI into creative resources and practical methodologies.

On a theoretical level, Park et al. (2023) demonstrated that GAI systems can exhibit human-like goal-directed behavior and offer valuable cognitive support to learners. Consequently, the core pedagogical purpose of experimental animation education is shifting from teaching students to operate digital tools to guiding them to build reflective, iterative and collaborative relationships with algorithmic systems. Related studies indicate that effective human-AI collaboration relies more on timely, complementary interaction mechanisms than on the capabilities of the tool itself (Bansal et al., 2019). Meanwhile, natural-language-driven programming exemplified by “Vibe Coding” lowers the technical barrier for art students entering dynamic visual systems. By enabling them to explore movement, interaction, and audiovisual logic through parameters, which aligns seamlessly with the non-linear and exploratory nature of experimental animation.

Addressing these dynamics, this study develops a partner-oriented human-AI collaborative pedagogical framework to support creative collaboration between students and GAI. This research addresses two research questions:

RQ1: How can a human-AI collaborative pedagogical framework that integrates GAI and Vibe Coding be effectively designed and structured for undergraduate experimental animation education?

RQ2: How does this framework prompt students to re-evaluate artistic intention, motion, media, and authorship in experimental animation, and how does it facilitate their transition from instrumental AI tool use toward critical guidance and collaborative creation?

To answer these questions, this study adopts design-based research and follows an iterative logic of design, implementation, reflection, and redesign. Implemented within a five-week course, the resulting “generation-feedback-evolution” framework connects concept development, visual experimentation, motion translation, and final integration. Drawing on students’ creative process portfolios and questionnaires administered at three stages of the course, the analysis examines how AI’s generativity, unpredictability, and iterability can be harnessed as creative resources for media experimentation and authorial expression.

## 2 Literature review

Research on AI in creative education has gradually transitioned from a focus on mere tool-based efficiency toward deeper examinations of collaboration, cognitive support, and creative subjectivity. This transition has prompted vibrant empirical discussions on the co-creative potential of algorithms, the dynamics of human-AI collaboration, and the preservation of creative agency. Yet, the development of this shift unfolds unevenly, presenting distinct trajectories and pedagogical challenges across varying technological modalities and disciplinary contexts.

### 2.1 Generative artificial intelligence in art education

The conceptualization of “AI as a partner” traces back to Lubart’s (2005) seminal taxonomy of four roles computers can play as human partners in the creative process (Lubart, 2005), a framework that has been revitalized in the GAI era as a manifesto for human-AI collaborative creativity (Vinchon et al., 2023). Existing empirical studies show that AI-assisted co-creation can significantly enhance the development of students’ creative fluency, flexibility, and elaboration (Zhang et al., 2025). Moreover, it provides evidence-based, process-oriented feedback (Meyer et al., 2024), and reduces cognitive burden under certain conditions to enhance learning efficiency (Dong et al., 2025). Through a systematic review of 36 empirical studies, Romero and Urmeneta (2026) classify AI’s roles into three categories: a facilitator of human-led creativity, a co-creative partner, and a semi-autonomous content generator, thereby highlighting the critical importance of “co-creative AI literacy.” However, while GAI introduces efficiency and novelty, it simultaneously risks undermining students’ design thinking and cognitive capabilities, resulting in formulaic and convergent design outputs (Hou et al., 2025), producing “polished mediocrity” that compromises the subjectivity of the creative work (Li and Liu, 2025). Consequently, design education must prioritize the cultivation of prompt literacy and AI visual literacy, ensuring that students can direct AI generation through critical thinking and distinctive narratives (Hwang and Wu, 2025). This shifts the pedagogical focus away from esthetic and formal “outputs” toward “outcomes” centered on critical thinking, collaboration, and iterative problem-solving (Rowe, 2025).

Concurrently, the paradigms of human-AI collaboration are also being continuously reshaped by technological evolution. In 2025, Karpathy introduced the concept of “Vibe Coding” to describe a programming practice in which natural-language intent drives AI to generate, debug, and iterate code. Sarkar and Drosos (2025) later characterized this practice as “programming through conversation,” noting that it redistributes cognitive labor between humans and machines while lowering technical barriers. Integrating this concept with flow theory, Royal (2026) proposed an AI-augmented coding education model, validating its potential to democratize access to programming education. Overall, current research remains concentrated within technical domains such as programming and computer science education. The application of Vibe Coding in art education—particularly in experimental animation pedagogy—remains unexplored, with limited research addressing the synergistic mechanisms between generative image systems and AI-assisted programming.

### 2.2 Technical transformation and practical challenges in experimental animation pedagogy

Experimental animation is fundamentally characterized by exploration, abstraction, and authorial subjectivity (Russett and Starr, 1976). From direct animation, abstraction, and visual music to computer-generated and algorithmic images, its evolution has always been deeply intertwined with artistic innovation and technological shifts. Rather than a linear “execution” of a preset plan, experimental animation production is an iterative process of continuous discovery, adjustment, and emergent generation (Taberham, 2019). Consequently, evaluating the utility of GAI in experimental animation pedagogy must not be evaluated only by efficiency; instead, it must assess the technology’s capacity to support open-ended exploration, media experimentation, and the cultivation of a reflective authorial consciousness.

Existing literature indicates that GAI can improve learning outcomes, knowledge transfer, and practical application in animation education. For instance, platforms utilizing generative adversarial networks (GANs) and variational autoencoders (VAEs) enable style-transfer practices that deepen students’ comprehension of stylistic features and esthetic paradigms (Yao et al., 2025). Concurrently, as animation media expand from traditional paper and physical models to dynamic moving images and transmedia narrative, AI’s intervention necessitates a fundamental re-evaluation of curriculum design, pedagogical models, and creative workflows (Tang et al., 2022). However, the integration of GAI also poses significant challenges. Its systematic reliance on pre-existing algorithms and historical datasets threatens to diminish contingency, experimental affect, and authorial lived experience, ultimately, leading to stylistic homogenization (Liu, 2023). This outcome stands in direct opposition to the core tenets of experimental animation: authorial subjectivity, media reflexivity, and productive unpredictability.

Thus, current applications of AI in experimental animation pedagogy remain largely confined to the functional integration of isolated tools. They have yet to fully address the field’s demands for exploratory inquiry, cross-modal translation, and critical iteration. To address this gap, there is a clear necessity to construct a pedagogical framework that integrates GAI and Vibe Coding, allowing for a structured investigation into how both technologies intersect with students’ concept development, motion translation, work revision, and authorial judgment.

## 3 Pedagogical framework design

**Figure 1:**
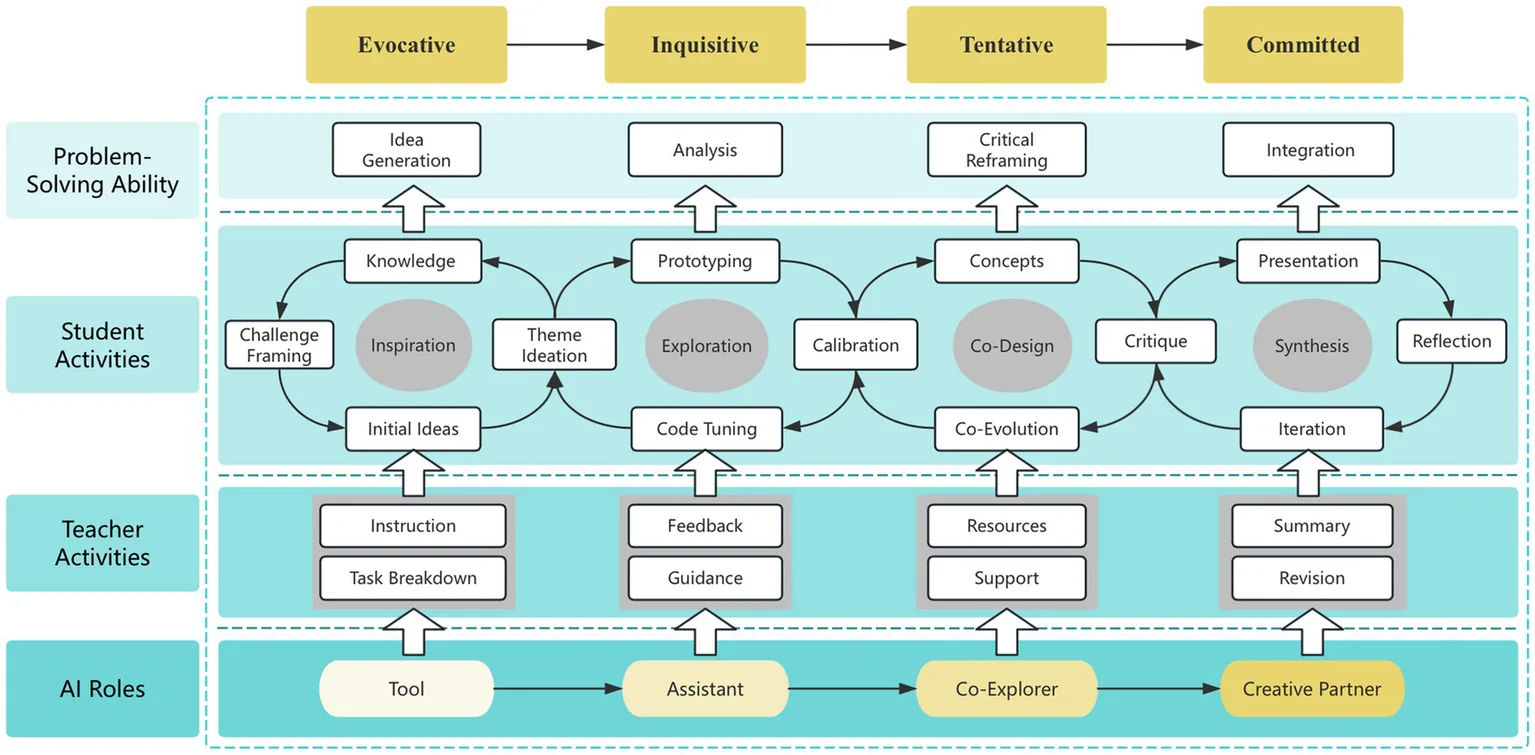
Human-AI collaborative framework for experimental animation pedagogy: From tool to partner.

To address the aforementioned research questions, this study proposes a human-AI collaborative framework tailored for experimental animation pedagogy (Figure 1). Driven by a core cycle of “generation-feedback-evolution,” the framework structures the students’ creative journey from initial concept development to final animation production. Unlike a linear “command-execution” model, it positions the human-AI relationship as a reflective, negotiable, and deeply collaborative relationship.

The framework consists of four distinct, interlocking structural layers.

The first layer outlines the four stages of the pedagogical process, mapping onto the complete workflow of experimental animation from conceptual divergence to final production. The first stage, “evocative,” focuses on inspiration and introductory scaffolding to help students construct foundational disciplinary knowledge and critical problem awareness. The second stage, “inquisitive,” guides students in deploying GAI and vibe coding across iterative generation cycles, leveraging algorithmic unpredictability (loss of control) as a catalyst for creative expansion. The third stage, “tentative,” emphasizes negotiation and evolution, prompting students to gradually solidify their creative direction through human-AI dialog and self-reflection. The fourth stage, “committed,” directs students toward final synthesis and contextualization, where they complete the loop from design thinking to design practice by compiling their animated shorts and drafting human-AI collaboration statements.

The second layer represents a four-dimensional progression of problem-solving capabilities. Throughout the four co-creative stages, students develop four interconnected, progressively deepening cognitive dimensions: creative divergence, analysis and expression, critical reconstruction, and complex integration.

The third layer comprises open-cycle learning activities. Initiated by acquiring new knowledge, decoding the prompt, and establishing thematic intent, students transition through prototype generation, feedback calibration, prompt and code refinement, proposal evaluation, and project redesign. Concurrently, the instructor’s role shifts from a conventional transmitter of knowledge to an organizer of design thinking and a facilitator of critical inquiry.

The fourth layer captures the evolution of AI’s collaborative modality and role perception. Rather than occurring spontaneously, this role transition is gradually constructed through pedagogical dynamics, task constraints, and student engagement patterns. In the initial phase, the AI serves primarily as a command-responsive tool. By the final phase, students are expected to treat generative outputs and code-driven motion as objects of critique, negotiation, and modification. Therefore, the developmental trajectory of “tool—assistant—exploratory resource—creative collaborator” precisely characterizes the shift in student usage patterns and cognitive perception of the technology.

## 4 Research method

### 4.1 Research context and participants

This study was conducted within a five-week Experimental Animation course offered to third-year undergraduate students majoring in Digital Media Art. The participant cohort consisted of 40 students who possessed a foundational background in traditional animation. All participants had completed prerequisite coursework covering basic animation principles and production workflows, and thus possessed standard digital tool literacy. However, none of them had prior experience with AI-assisted tools or vibe coding.

### 4.2 Design-based research process

**Figure 2:**
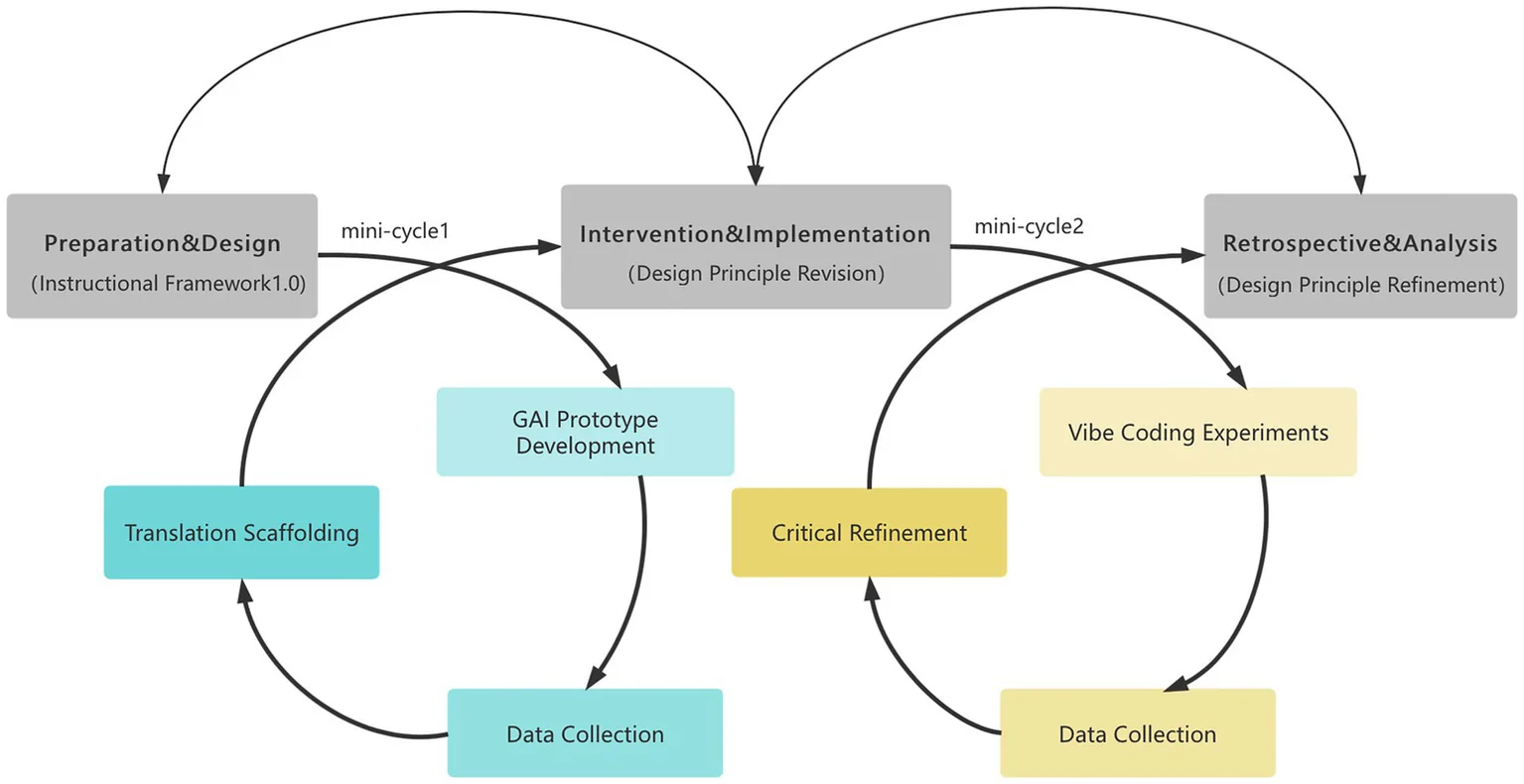
Iterative DBR cycle of the human-AI collaborative teaching framework.

Utilizing a Design-Based Research (DBR) methodology, this study investigated the practical execution of the proposed pedagogical framework within an authentic classroom setting through iterative cycles of design, implementation, analysis, and redesign (Collins et al., 2004). The five-week course was structured as a single, comprehensive DBR cycle consisting of three main phases: preparation and design, intervention and implementation, and review and analysis. To ensure responsiveness, two mini-iterations were embedded between these phases, allowing the instructional content to be dynamically adjusted based on real-time analysis of the students’ processual artifacts (Figure 2).

Guided by the aforementioned methodology, the course was themed “The Life of Objects: Reconstructing Images from Non-Human Perspectives.” Students were asked to observe everyday objects, develop a non-human narrative perspective, generate visual concepts through GAI, and subsequently translate these concepts into experimental moving images using Vibe Coding alongside other animation techniques. The five-week course followed a micro-cycle of “concept generation—motion translation—comprehensive reflection,” establishing an observable, analyzable, and iterative pedagogical workflow (Table 1).

**Table 1:** Simplified course content and instructional design.

Period	Stage	Objective	Activities	Data collection
Week 1	Evocative	Learn basic GAI knowledge and begin initial design.	1. Introduce the theme “The Life of Objects: Reconstructing Images from Non-Human Perspectives.” 2. Explain GAI basics and practice simple prompting. 3. Select objects and create initial sketches.	Initial descriptions; class observations
Week 2	Inquisitive	Build an initial human-AI dialog workflow	1. Use GAI to expand sketches into conceptual images. 2. Conduct dynamic translation practices, explore non-human perception.	Process log 1; revised drafts
Interim Period	Mini-iteration 1	Review early data; track student–AI interaction	Analyze process logs and drafts; adjust translation paths and creative strategies	Reflection journal 1
Week 3	Tentative	Combine GAI and Vibe Coding for project work	1. Introduce Vibe Coding for dynamic image and sound processing. 2. Create dynamic experimental fragments using GAI materials.	Process log 2; experiment samples
Week 4	Tentative	Strengthen critical evaluation; understand human–AI roles	1. Revise prompts or Vibe Coding scripts. 2. Guide critical selection and refinement of AI-generated outcomes.	Midterm report; informal critique
Interim Period	Mini-iteration 2	Review recent data; track critical engagement	Analyze logs and experiment data to strengthen critical collaboration.	Reflection journal 2
Week 5	Committed	Produce and present final short animation	1. Complete final piece and present in critique session. 2. Write a collaboration statement reflecting on concept, process, and authorship.	Final work; final reflection

### 4.3 Data collection and analysis

Data collection spanned the whole instructional cycle, triangulating qualitative processual artifacts with quantitative survey data. The qualitative data comprised student process logs, prompt iteration histories, sketches, generated images, motion clips, reflective journals, and project statements. These materials were analyzed to trace shifts in students’ capabilities regarding intention structuring, motion translation, critical iteration, and complex integration. Furthermore, purposive case studies were utilized to illustrate how students transitioned from instrumental tool operation to collaborative, co-creative practices.

The quantitative phase employed a three-wave longitudinal survey administered at Week 1, Week 3, and Week 5. Utilizing a five-point Likert scale, the instrument consisted of a six-item “AI Partner Perception Scale” and a 10-item “Cognitive Load Scale.” The former measured whether students regarded AI as a collaborative partner that facilitates inspiration, feedback, and negotiation. The latter, adapted from Leppink et al. (2013), measured three dimensions of cognitive load: intrinsic load (reflecting task complexity), extraneous load (reflecting the mental burden imposed by technological and tool-based barriers), and germane load (reflecting the cognitive resources dedicated to deep learning, creative comprehension, and critical decision-making).

For data analysis, reverse-coded items were first recoded, and dimension means were calculated. Internal consistency was evaluated using Cronbach’s alpha, and the normality of the paired difference scores was assessed via P–P plots. Differences across the three time points were evaluated using paired-samples t-tests per dimension (*N* = 40, *df* = 39 for each comparison). Cohen’s *d*_z_ was calculated as the mean paired difference divided by the standard deviation of the paired differences ( $d_{z}=t/\surd N\;).$ No multiplicity adjustment was applied; therefore, all reported *p*-values are unadjusted and should be interpreted as exploratory rather than confirmatory. Pearson’s correlation analysis was additionally conducted to examine the relationships between post-test AI partner perception scores and the three cognitive load dimensions. Given the absence of a control group, the quantitative results describe only within-group changes over time and associations among variables. These results were synthesized with the qualitative data to provide a holistic interpretation without implying strict causality.

## 5 Findings and discussion

### 5.1 Quantitative results: within-group changes in student cognition and cognitive load

#### 5.1.1 Data quality validation

Reliability analysis of the questionnaire indicated that Cronbach’s alpha coefficients for all dimensions across the pre-test, mid-test, and post-test were above 0.70 (Table 2), indicating acceptable internal consistency. Analysis of the P–P plots and Harman’s single-factor test showed no significant deviations from normality or severe common-method bias. The first unrotated factor explained 39.914% of the variance, confirming that the data quality was statistically robust enough to support subsequent parametric analyses.

**Table 2:** Reliability coefficients.

Dimension	Pre-test	Mid-test	Post-test
AI partner perception	0.923	0.898	0.841
Intrinsic load	0.854	0.719	0.824
Extraneous load	0.701	0.896	0.778
Germane load	0.905	0.859	0.873

#### 5.1.2 Paired-sample t-tests at three time points

To evaluate changes in student perception and cognitive load midway through the course, paired-sample t-tests were first conducted on the pre-test and mid-test data. From the pre-test to the mid-test, all dimensions showed slight changes, but none reached significance. This suggests that in the first half of the course, students were primarily occupied with tool familiarization, prompt iteration, and preliminary motion translation, meaning that a stable, collaborative human-AI relationship had not yet formed (Table 3).

**Table 3:** Paired-sample t test: pre-test and mid-test.

Dimension	Pre-test	Mid-test	T	*p*	Cohen’s d
AI partner perception	3.30 ± 0.99	3.50 ± 0.75	−1.248	0.219	−0.197
Intrinsic load	3.42 ± 0.86	3.29 ± 0.63	1.088	0.283	0.172
Extraneous load	3.91 ± 0.54	3.62 ± 0.93	1.53	0.134	0.242
Germane load	3.38 ± 1.00	3.43 ± 0.87	−0.554	0.583	−0.088

A subsequent comparison of the pre-test and post-test data revealed that after the five-week pedagogical intervention, students’ AI partner perception, extraneous load, and germane load all experienced statistically significant changes, while intrinsic load remained stable (Table 4). This pattern suggests that the pedagogical intervention did not introduce additional technical stressors. On the contrary, students increasingly conceptualized AI as a collaborative resource; the additional burden from tool operation and code debugging decreased; and more cognitive resources were invested in creative understanding, critical evaluation, and meaning-making. These findings point to an apparent cognitive migration from mechanical tool use toward critical, creative integration.

**Table 4:** Paired-sample t test: pre-test and post-test.

Dimension	Pre-test	Post-test	T	*p*	Cohen’s d
AI partner perception	3.30 ± 0.99	4.09 ± 0.63	−4.177	<0.001	−0.660
Intrinsic load	3.42 ± 0.86	3.49 ± 0.83	−0.383	0.704	−0.061
Extraneous load	3.91 ± 0.54	2.90 ± 0.91	6.306	<0.001	0.997
Germane load	3.38 ± 1.00	3.97 ± 0.75	−2.74	0.009	−0.433

To determine which stage of the course drove these cognitive shifts, mid-test and post-test data were then compared. As shown in Table 5, from the mid-point to the end of the course, AI partner perception, extraneous load, and germane load all changed significantly, whereas intrinsic load exhibited no significant change.

**Table 5:** Paired-sample t test: mid-test and post-test.

Dimension	Mid-test	Post-test	T	*p*	Cohen’s d
AI partner perception	3.50 ± 0.75	4.09 ± 0.63	−3.473	0.001	−0.549
Intrinsic load	3.29 ± 0.63	3.49 ± 0.83	−1.162	0.252	−0.184
Extraneous load	3.62 ± 0.93	2.90 ± 0.91	3.407	0.002	0.539
Germane load	3.43 ± 0.87	3.97 ± 0.75	−2.698	0.01	−0.427

Specifically, AI partner perception increased from 3.50 ± 0.75 to 4.09 ± 0.63, representing a medium effect size. This pattern is consistent with the consolidation of students’ recognition of AI as an active creative partner occurring mainly in the latter half of the course. The lack of a significant change in intrinsic load suggests that students’ perception of the core task complexity did not rise. Concurrently, extraneous load dropped from 3.62 ± 0.93 to 2.90 ± 0.91, coinciding with a period of iterative hands-on practice, timely instructor interventions, and peer dialogs that may have contributed to easing technical and operational barriers. Finally, the significant increase in germane load is consistent with students redirecting their cognitive efforts toward deep creative reflection, critical judgment, and conceptual synthesis as the course progressed.

#### 5.1.3 Correlation analysis

Based on the post-test data, a Pearson correlation analysis was conducted across the four dimensions (Table 6).

**Table 6:** Correlation analysis.

Dimension	AI partner perception	Intrinsic load	Extraneous load	Germane load
AI partner perception	1			
Intrinsic load	0.602**	1		
Extraneous load	−0.563**	−0.450**	1	
Germane load	0.344*	0.379*	−0.414**	1

*p < 0.05, **p < 0.01.

“AI partner perception” exhibited a significant, moderate positive correlation with “intrinsic load” (*r* = 0.602, *p* < 0.01), a significant, moderate negative correlation with “extraneous load” (*r* = −0.563, *p* < 0.01), and a significant, weak positive correlation with “germane load” (*r* = 0.344, *p* < 0.05). Higher AI partner perception was associated with greater awareness of task complexity, lower levels of technical hindrance, and greater cognitive resources dedicated to productive learning and reflection. Additionally, “extraneous load” was significantly and moderately negatively correlated with “germane load” (*r* = −0.414, *p* < 0.01), pointing to an association between lower operational burden and increased cognitive bandwidth available for creative evaluation and problem-solving.

In summary, while the pedagogical framework did not reduce the inherent complexity of the experimental animation task, extraneous load decreased significantly, and AI partner perception and germane load both increased significantly. However, because quantitative data only capture aggregate, longitudinal trends, they cannot fully capture how these cognitive shifts unfolded during the creative pipeline. To address this, the subsequent section synthesizes qualitative evidence—including student process logs, prompt iteration histories, animation test clips, reflective diaries, and final projects—to analyze these transformations across three analytical levels: conceptual expression, the reconstruction of authorship, and complex integration.

### 5.2 Conceptual expression: from image commands to cross-modal translation

In the first half of the course, students’ creative process shifted from a passive quest of acquiring raw image outputs to an active organization of conceptual expressions. Students used GAI to generate diverse creative options, translating abstract ideas into text prompts the machine could interpret, and attempting to bridge the gap between static image generation and temporal, dynamic expression. At this stage, prompts served as both input commands and mediators linking the artist’s intent, the system’s output, and subsequent edits. By analyzing, adjusting, and curating these outputs, students gradually clarified their core concepts.

Grounded in this concept, the course first guided students to observe everyday objects, make sketches, gather reference material, and draw rough drafts. Students then translated their personal observations into language and used GAI tools for visual ideation. The case studies in Table 7 show that students’ prompts evolved from simple visual descriptions into rich, structured expressions containing detailed spatial, emotional, symbolic, and narrative information. Students began to treat their text-based dialog with AI as a systematic tool for exploration, choice-making, and conceptual clarity, rather than a transaction of one-way commands.

**Table 7:** Relationship between prompt iteration and cognitive development.

Sample	Prompt 1	Prompt 2	Reflection
Student A	“Generate a lonely apple based on my sketch, rendered in a surrealist style.”	“Show the apple’s inner cellular nucleus, where an intertwined grid network encapsulates the apple’s memories, symbolizing the absolute infinity of its interior space.”	I realized I cannot rely on common themes or simple visual cues. I need varied, precise descriptions and narrative details to help AI better interpret my ideas.
Student B	“Generate a longevity spiral egg from my reference image and explore different interpretations.”	“A longevity spiral egg with strong reproductive and destructive energy; its unstable lines imply internal tension and fear.”	When my prompts were vague, AI repeated my original design. To avoid uninteresting loops, I added sensory details and searched for more references to guide AI toward more creative and diverse outputs.

**Figure 3:**
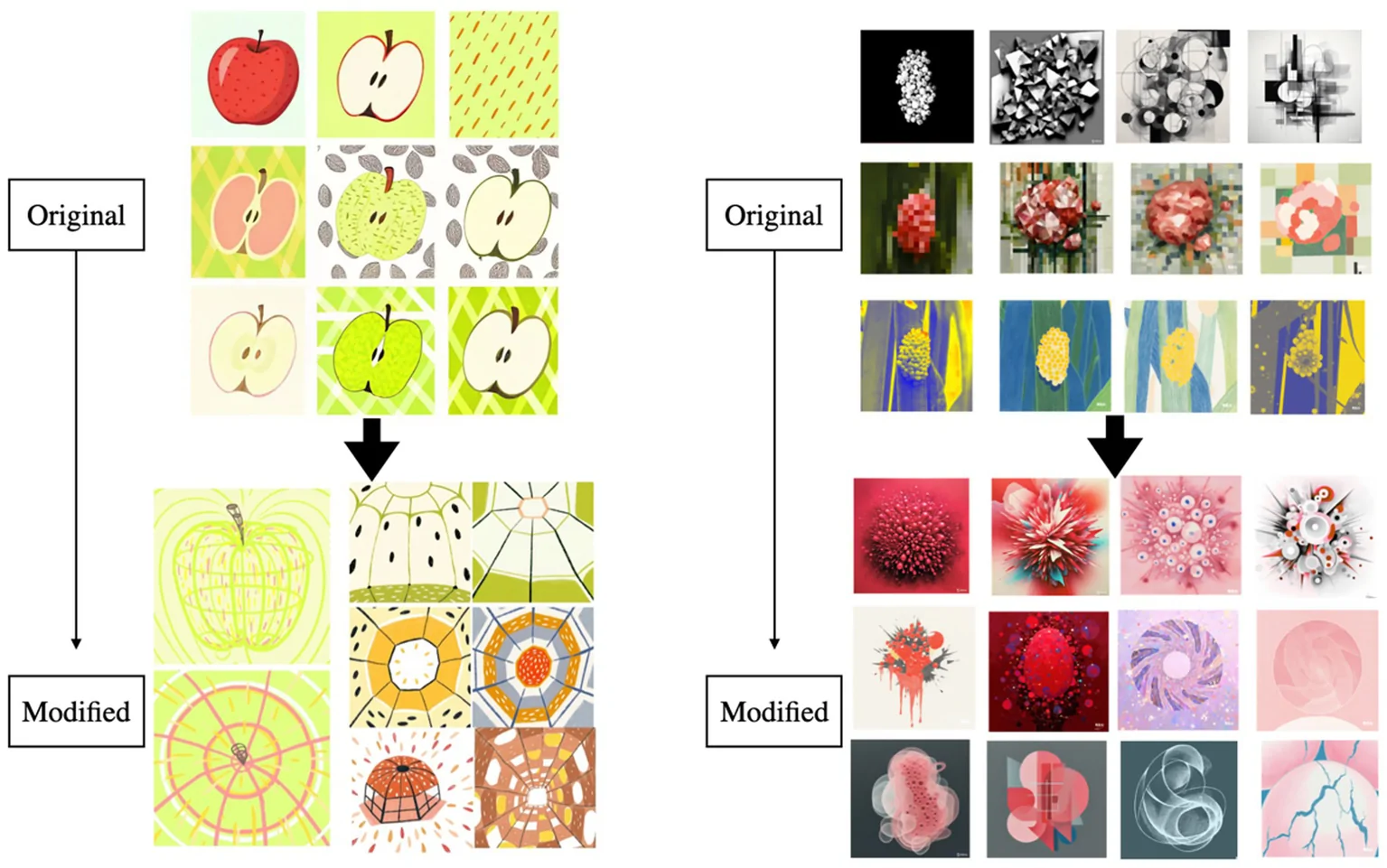
Image changes produced by prompt iteration.

Through sustained prompt iteration and dialog, students yielded highly evocative creative ideas, setting the esthetic and conceptual tone for their final animation pieces. As shown in Figure 3, the evolution of these prompts was not merely a matter of adding adjectives; it proved that the students’ creative intentions had evolved from vague thoughts into clear, adjustable, and translatable concepts. This shift allowed students to organize their ideas more precisely and take the lead in guiding the GAI to stretch their visual boundaries.

**Figure 4:**
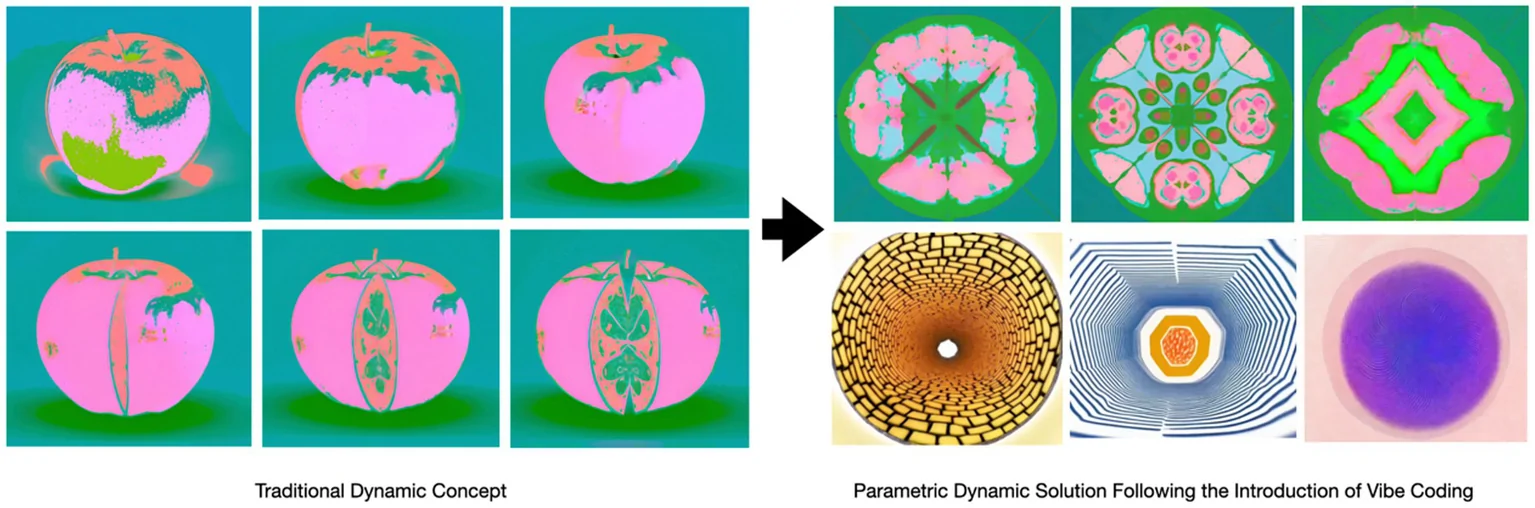
Comparison of dynamic translation approaches.

When the course advanced to the dynamic-expression stage, students needed to transform static images into theme-driven experimental moving images. Difficulties in cross-modal translation quickly emerged. Dynamic descriptors within prompts—such as “loop,” “diffuse,” or “breathe”—were frequently flattened by generative systems into rudimentary scaling, drifting, or morphing behaviors, failing to manifest the intended non-human sensory logic (Table 8). This widespread challenge created a crucial pedagogical opening for the intervention of Vibe Coding. Consequently, the IDE platform Cursor was integrated into the syllabus to guide students in constructing generative motion mechanics via physical simulations and algorithmic parameters, enabling them to explore kinetic expression from a non-anthropocentric perspective. Figure 4 presents frame-by-frame screenshots extracted from the students’ dynamic outputs before and after the introduction of Vibe Coding. In the left panel, each frame was generated via simple rotation and scaling, resulting in only minor variation across the sequence. This visual similarity is not a sampling artifact; it reflects the limited kinetic vocabulary available before Vibe Coding was introduced. By contrast, frames in the right panel were created using parametric, code-driven motion, yielding substantially greater variation in trajectory, rhythm, and form. This contrast makes visible the qualitative shift enabled by Vibe Coding–from repetitive, tool-constrained motion to a generative range of kinetic possibilities. Vibe Coding effectively shifted students’ mindset from “how to animate” to “how to program a kinetic logic for an artistic concept.” In this workflow, GAI managed the generation of visual concepts, while Vibe Coding translated them into adjustable, dynamic kinetic systems.

**Table 8:** Students’ bottlenecks in dynamic generation.

Sample	Prompt	Output	Reflection journal
Student A	“Based on this image, create a slow, breathing-like motion inside the apple, similar to a cinematic effect.”	Only slight zooming and panning occurred, failing to produce a motion form that matched the apple’s internal breathing.	I wanted an abstract, non-human motion, but did not know how to express it. The AI only generated slow movement and scaling, which undermined the spatial depth of the image.

### 5.3 Reconstructing authorship: from accepting outputs to critical direction

**Figure 5:**
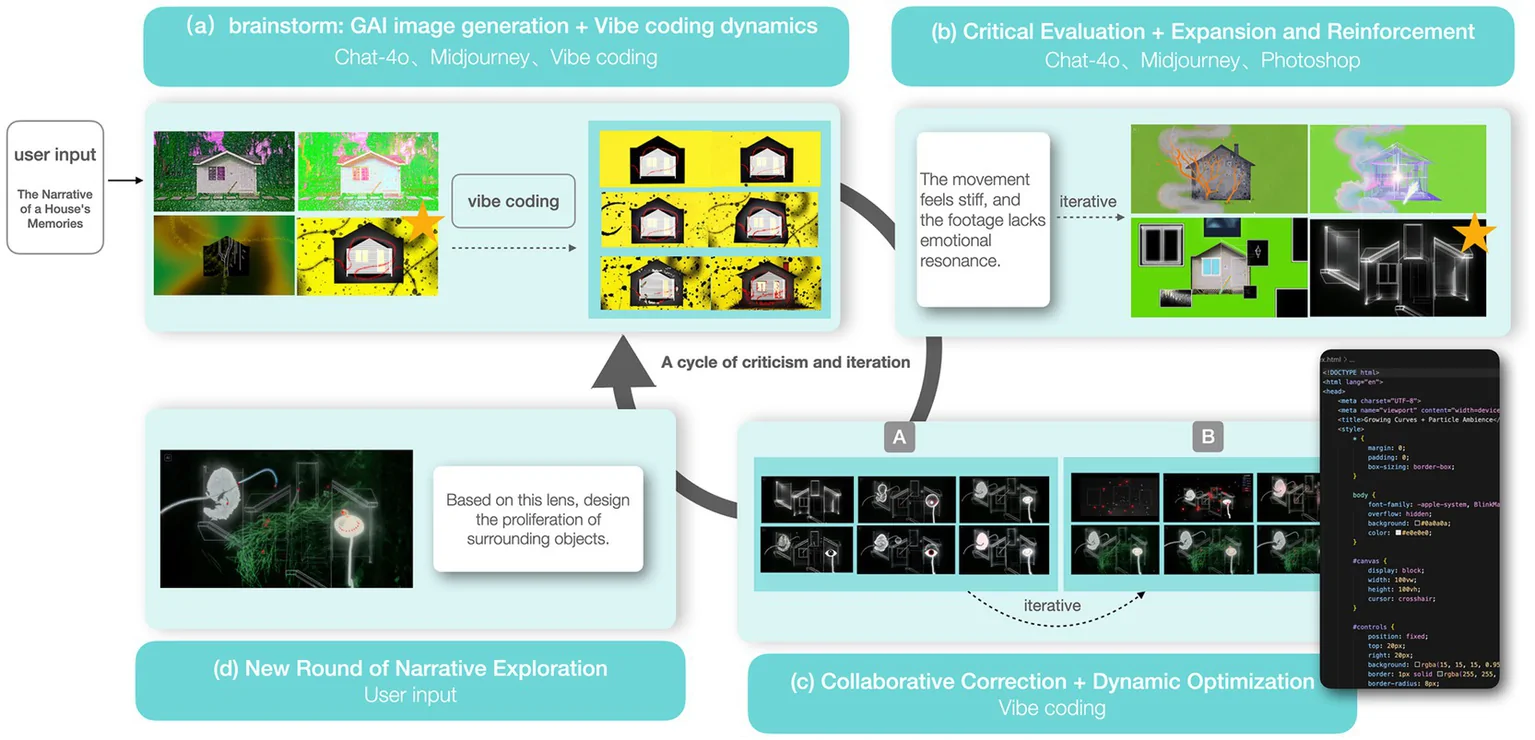
Critical iteration and collaborative revision process: **(a)** Brainstorm: GAI image generation and vibe coding dynamics; **(b)** critical evaluation, expansion, and reinforcement; **(c)** collaborative correction and dynamic optimization; **(d)** new round of narrative exploration.

During the advanced phase of the course, students’ core task transitioned to the narrative construction of their experimental animated shorts. Grounded in their initial design proposals, students began generating specific shot sequences and organizing a non-human narrative framework through diverse audiovisual techniques. They explored how cinematic structure and the gaze could externalize the “phenomenology of the object,” thereby interrogating the fundamental nature of perception itself. In this process, students needed to select appropriate technical trajectories tailored to their specific themes, continuously refining generative outputs to maintain narrative coherence, emotional resonance, and a non-human perspective. Under this paradigm, creation was no longer a pursuit of a one-time final output, but a process of selection, judgment, revision, and processing (Figure 5).

Throughout this process of critical iteration, students deepened their understanding and expression of creative concepts through ongoing dialog with AI systems. They shifted from mere technical execution to critically examining narrative architectures, rhythmic pacing, and media relationships. Technical output was no longer treated as the endpoint of creation but rather as highly malleable creative material open to interrogation, critique, and structural transformation. The creative frictions—manifested as gaps between generated imagery and artistic intent, or between kinetic concepts and underlying code logic—compelled students to maintain conceptual control across multiple interlocking technical systems. Consequently, authorial agency was not weakened by AI intervention; rather, it was actively reassembled through student judgment, selective curation, and transformation, manifesting as a sophisticated capacity to orchestrate generative systems, dynamic structures, and audiovisual materials (Table 9).

**Table 9:** Students’ reflections on collaboration with AI.

Sample	Reflection
Student D	Every AI-generated result became a starting point for dialog rather than an end. When I questioned whether the outputs fit my core idea or adjusted Vibe Coding parameters for precision, I realized I was no longer “using tools,” but negotiating with two creative partners with distinct strengths.
Student F	I began to look forward to AI responses, as they often revealed possibilities I had not considered. GAI and Vibe Coding felt like collaborators requiring different modes of communication. My role was to refine the central concept, pose clearer questions, and guide them toward more precise outcomes.
Student H	A major breakthrough in the second iteration was realizing that dissatisfaction with AI output did not mean abandoning it, but refining prompts and parameters to better express the intended emotion. Through this negotiation, the three of us formed a shared creative network.

### 5.4 Complex integration: from technical assistance to collaborative creation

**Figure 6:**
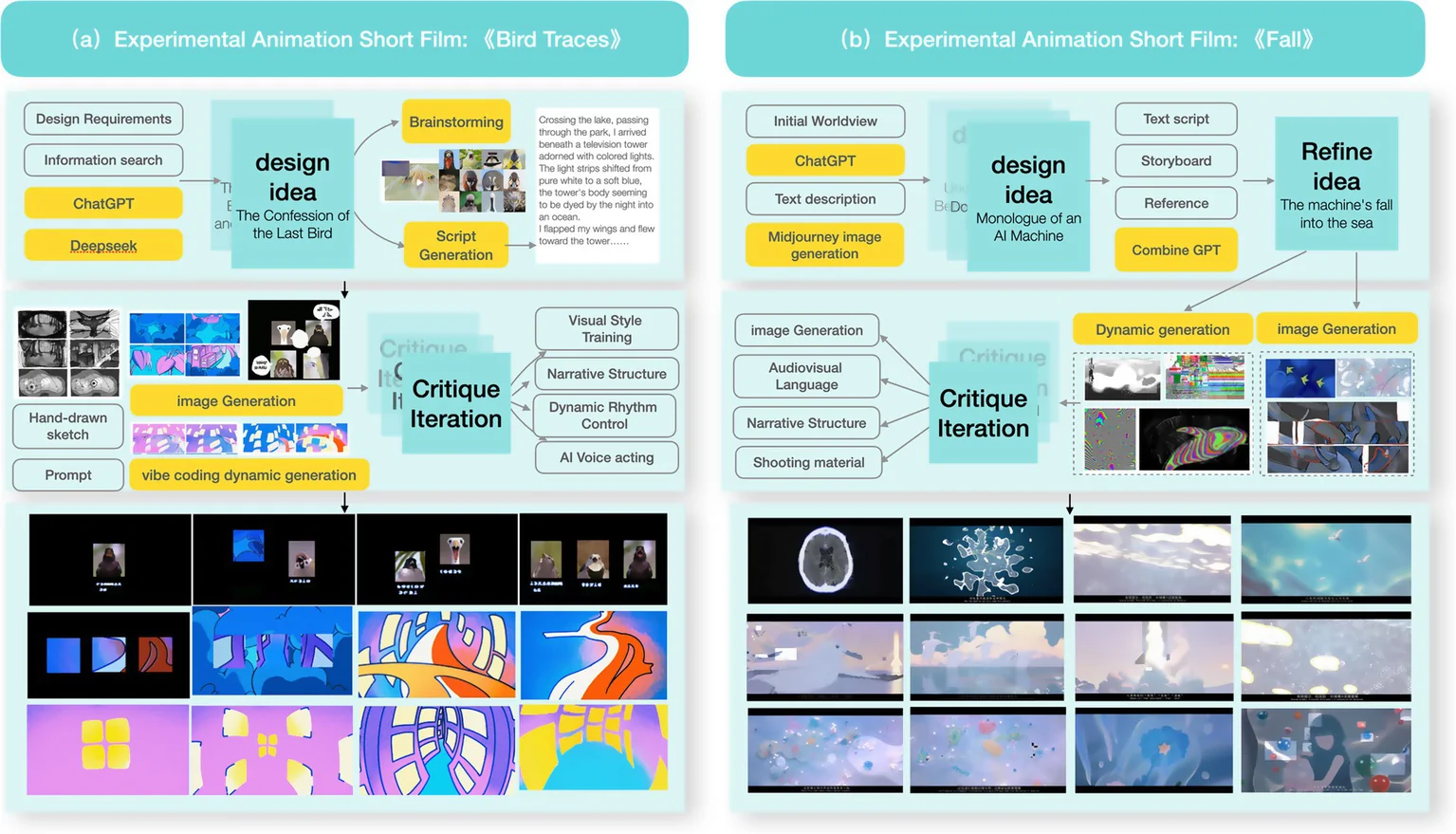
Student short-film works: **(a)** experimental animation short film *Bird Traces*; **(b)** experimental animation short *film Fall*.

In the final stage of the course, students shifted their focus toward integrating generated images, kinetic parameters, soundscapes, editing rhythm, and narrative structure. They had to select, refine, and reorganize large amounts of generated content to complete a short film and a creative statement. Different student groups developed different technical routes. This diversity was not only about tool choice; it also reflected how each group conceptualized the act of “experimentation.” The two projects in Figure 6 represent two experimental paths: one centered on perspective, the other on the medium itself. The first piece builds its visual world entirely from a bird’s-eye view, utilizing multiple parallel narratives. The viewpoint constantly shifts among soaring, perching, and hiding—intentionally moving away from ordinary human vision to make the gaze itself an object of critique. The second piece explores the concept of machine perception. It juxtaposes generative visual outputs, real-time visualization data, vibe coding, and stop-motion techniques to present a machine’s gradually dissolving self-awareness through an unstable, flickering visual vocabulary. Although the two projects followed different routes, both returned to the essence of experimental animation: embracing unstable, unpredictable forms to conduct artistic research, guided at every turn by the artist’s personal choices.

These practices show that AI did not replace students in production. Instead, it acted as a catalyst that kept media questions alive throughout the creative process. As an important part of a hybrid creative workflow, AI participated in a collaborative relationship beyond the traditional tool paradigm. This cooperative mode significantly expanded students’ creative thinking, turning linear, preset execution into a wide-open process of shared exploration, while deeply reinforcing their reflective and experimental spirits.

These practical students’ cases also offer concrete evidence for understanding the role of AI in creation. Creativity does not belong solely to a single human author; it can spark from the collective interaction between computational systems, individual creators, and human-agent teams (Maher, 2012). If AI is treated merely as a tool, human-AI collaboration becomes nothing more than a linear transaction of commands: the student sets a goal, the AI completes it, and the ultimate value is limited to speed and efficiency. However, experimental animation is inherently non-linear and full of uncertainty. In this course, the AI’s most profound role was serving as a source of feedback, a trigger for variations, and a medium for motion translation, constantly challenging students to rethink and modify their initial thoughts. Students did not just type out dictations; instead, they entered an open, shared creative space with the AI, collaborating through keywords, mood descriptions, reference styles, and generative feedback.

**Figure 7:**
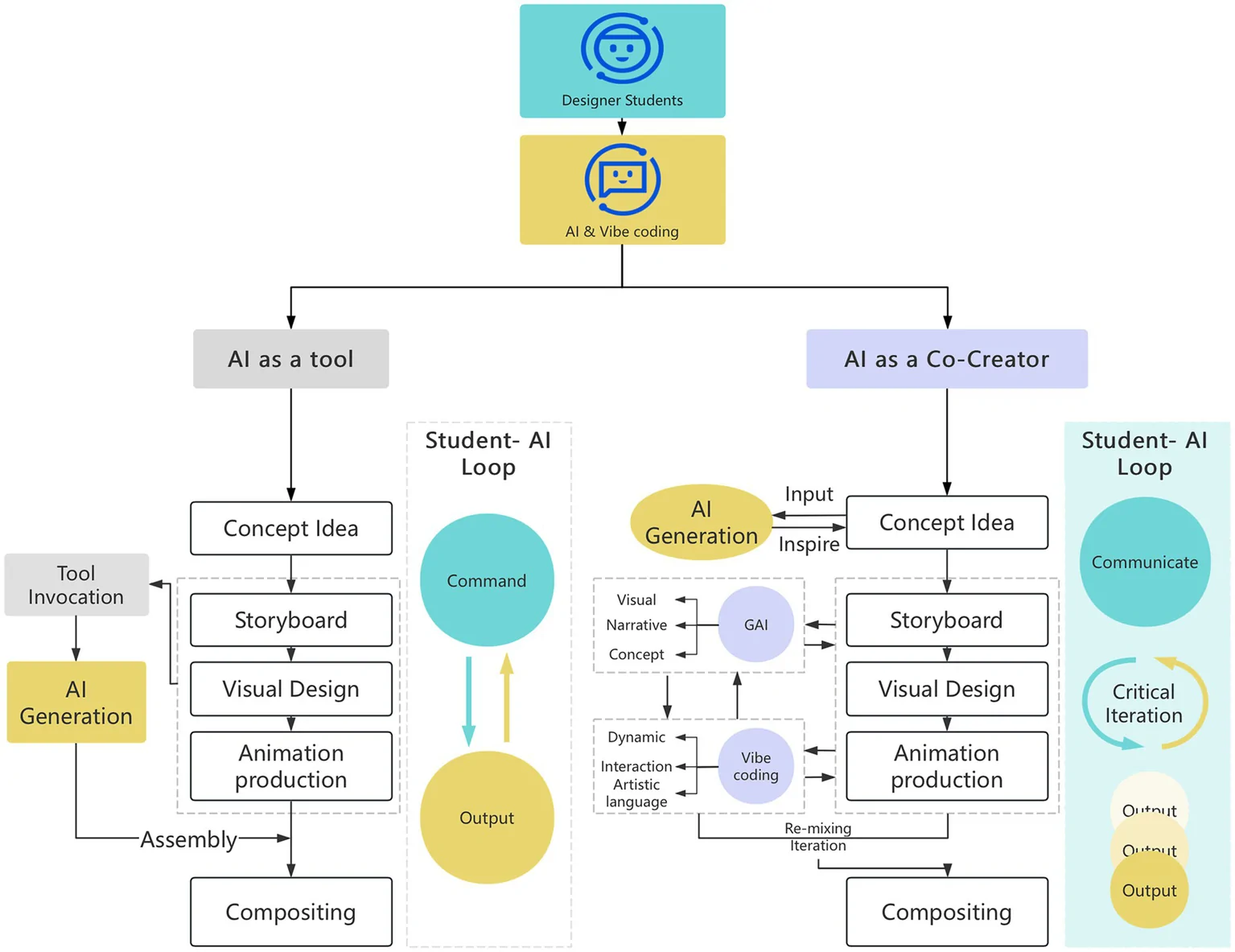
Comparison of two AI collaboration models in experimental animation production.

This interaction captures the core worth of GAI and Vibe Coding as true creative partners. They not only optimized the creative path but also expanded students’ thinking, making creation a process of discovery with intelligent agents (Figure 7). This represents the central ambition of this study: showing how an AI-driven classroom can guide students to discover personalized paths of human-machine collaboration. By learning to independently coordinate multiple technical tools under a unified artistic concept, students gradually construct their own creative methodology. What students developed was not merely software dexterity, but a robust collaborative capacity to face unpredictable outputs, make critical esthetic choices, and successfully achieve cross-media integration.

## 6 Conclusion

This study demonstrates that within undergraduate experimental animation education, the synergistic integration of GAI and Vibe Coding represents far more than a mere upgrade in production efficiency. Rather, it serves as a vital catalyst for transforming students’ creative practices. The proposed “generation-feedback-evolution” framework exhibits clear potential in guiding students away from instrumental tool deployment toward genuine co-creation. In the course, students’ creative process shifted from intention structuring and cross-modal translation to critical iteration and media integration. Consequently, students shifted from passive executors of algorithm outputs to active creative agents capable of organizing, evaluating, and directing diverse technical resources.

This transition underscores that “AI as a partner” is not an inherent affordance of the technology itself; it is a collaborative relationship constructed through structured pedagogical tasks, iterative feedback loops, and students’ authorial judgment. While AI can generate creative variance and provide feedback, it also carries an inherent tendency toward algorithmic patterning and cognitive inertia. Therefore, AI must neither be mythologized as an autonomous artistic subject nor simplified as a fully neutral tool. Human-AI collaboration can only serve experimental animation pedagogy when students treat AI outputs as negotiable creative materials that can be actively debated, revised, and restructured—all while maintaining critical control over concepts, media materiality, and ethical implications.

Accordingly, experimental animation pedagogy in the AI era must transcend basic software training and efficiency optimization. Educators should guide students to develop critical curation, cross-media integration, and authorial discernment amidst unpredictable technical feedback, transforming technological generation from an efficiency tool into negotiable artistic partners. The collaborative use of GAI and Vibe Coding offers a generative educational landscape and a practical trajectory for re-evaluating the intersections of creative intention, motion language, media relations, and authorial awareness in experimental animation.

## Data Availability

The raw data supporting the conclusions of this article will be made available by the authors, without undue reservation.
